# Comparative Analysis of Deformation Determination by Applying Fiber-optic 2D Deflection Sensors and Geodetic Measurements

**DOI:** 10.3390/s19040844

**Published:** 2019-02-18

**Authors:** Marko Z. Marković, Jovan S. Bajić, Mehmed Batilović, Zoran Sušić, Ana Joža, Goran M. Stojanović

**Affiliations:** 1 Department of Civil Engineering and Geodesy, Faculty of Technical Sciences, University of Novi Sad, Novi Sad 21101, Serbia; mehmed@uns.ac.rs (M.B.); zsusic@uns.ac.rs (Z.S.); 2 Department of Power, Electronic and Telecommunication Engineering, Faculty of Technical Sciences, University of Novi Sad, Novi Sad 21101, Serbia; bajic@uns.ac.rs (J.S.B.); anajoza@uns.ac.rs (A.J.); sgoran@uns.ac.rs (G.M.S.)

**Keywords:** fiber-optic curvature sensor, total station, deformation analysis, measurements, displacement

## Abstract

In the paper the description of an experiment for a comparative analysis of two different methods for deformation determination, geodetic and 2D deflection sensors based on fiber-optic curvature sensors (FOCSs) is given. The experiment is performed by a using specially designed assembly which makes it possible to apply both methods. For performing geodetic measurements, a geodetic micro-network is established. Measurements by applying a 2D deflection sensor and three total stations are carried out for comparison. The data processing comprises graphical and numerical analysis of the results. Based on the presented results the potential of 2D deflection sensor application in structural health monitoring (SHM) procedures is indicated. The analysis of the measurement results also indicates the importance of integrating various types of sensors for obtaining more accurate and more reliable deformation measurements results.

## 1. Introduction

Civil engineering objects (bridges, tunnels, dams, etc.) are exposed to deformations under the influence of various factors, such as changes of ground water level, tectonic phenomena, landslides, etc. The development and integration of interdisciplinary methods of measuring and data processing have undergone a revolutionary transformation from the mere noting and describing a deformation towards the analysis of what causes the phenomenon of deformations. There are several measurement methods aimed at deformation detection. They can be divided into geodetic and non-geodetic methods.

The conventional geodetic measurements offer a high precision in the relative positioning of the discrete (control) points and yield a global picture of deformations affecting the object under observation. However, such measurements are slow and their adaptation to continuous and automatic monitoring is complicated and relatively expensive [1].

Geotechnical sensors provide exceptionally precise information about deformations and they are easily adapted to continuous, completely automatic and telemetric data acquisition. Compared to conventional and satellite geodetic methods, geotechnical sensors are mostly independent of some outdoor conditions, such as snow cover and weak visibility. However, the information provided by them is only local, concerning discrete points [1].

The geodetic methods are based on establishing a geodetic micro-network of points which are related through different types of measurements (measurements of angles, lengths, altitude differences, GNSS vectors). In order to calculate statistical quality estimation and errors identification, measurements are performed with optimal redundancy. By applying such methods, global information about the behavior of an engineering object exposed to deformations can be obtained. Short periodic measurements of objects (dams, bridges, etc.) deformations by geodetic methods are performed based on points of the geodetic micro-network. This concept is also shown in this paper.

Depending on the type of optical fibers used and the change of the optical signal properties due to environmental influences, there are various fiber-optic sensor (FOS) configurations. In practice, the most widely used FOS are based on one of the four principles: change of light intensity (intensiometric FOS), spectrum, polarization or phase (interferometric FOS) [2,3]. In all four cases the change of physical quantity interacts with the light in the optical fiber or with a non-fiber sensor which is connected to the optical fiber so that it can register changes of intensity, spectrum, polarization or phase [4]. The main advantages of most sensors based on FOS technology are the use of low-power energy sources, immunity to strong electromagnetic fields, corrosion resistance, small size, high sensitivity and large bandwidth.

Optical fibers with fiber Bragg gratings (FBGs) are increasingly being used as photonic sensors for various applications in SHM [4]. These highly precise systems require however expensive light sources and spectrum analyzers. Also, they cannot distinguish between concave and convex bending. In addition, their accuracy is affected by external factors, such as temperature, and because of that their application is limited [5]. The application of FBGs, similar to the 2D deflection sensor application in deformation measurement which will be explained in more detail in the following text, is described in [6]. Deformation measurements based on integrated geodetic and FBG sensors system are presented in [7].

Fiber-optic interferometric sensors and curvature analysis using parallel sensor topology are explained in details in [8,9,10]. The main advantage of interferometric FOSs is their great potential in practical applications, such as monitoring deformations of airplanes, ships and constructions in real time [10]. Nevertheless, these high-accuracy systems require relatively complicated measurement systems and because of that they are often considered expensive [5].

The FOSs may be designed so that they can discriminate in the spatial mode, and in this way, the measurand can be determined along the length of the fiber itself, in a process normally termed distributed sensing. This principle has been employed widely in the temperature measurement using non-linear effects in fibers, such as Brillouin or Raman scattering or in some types of strain sensing [11,12,13].

By using FOS based on a change of light intensity it is possible to measure bending deformations of the structure. Depending on the mechanical configuration of an intensiometric FOS many physical quantities, such as strain, torsion, position, can be calculated on the basis of bending measurements [14]. The sensitivity of an optical fiber to bending can be increased by applying various types of structural imperfections on the surface of an optical fiber. By applying such imperfections on the optical fiber, besides increasing the bending sensitivity, it is also possible to determine the bending direction (positive or negative) [15,16,17,18]. Also, in the case of FOCS a simple system for signal demodulation is sufficient, in contrast to expensive techniques of processing and analyzing signals from interferometric/polarimetric sensors with problems, such as signal fading, interrupt and sign ambiguity, nonlinearity and multi-valued response [19]. The FOCS operation principle is given in more detail in the study by Fu and Di [14].

The main characteristics of the 2D deflection sensor are the ability to monitor both static and dynamic deformations with high observation frequency, high resolution, accuracy and reliability of measurements, long-term preservation and stability, low cost and simple implementation. Compared to other similar sensor solutions, the 2D deflection sensor is characterized by a robust and low-cost design making it an economical and suitable solution for application in the SHM process. This is supported by the fact that to produce 2D deflection sensor robust and low-cost plastic (PMMA) optical fibers were used. In this paper, a new approach, developed for monitoring of engineering structures, is considered, which, in addition to geodetic measurements, applies 2D deflection sensor in detecting local deformations [20].

## 2. Geodetic Deformation Analysis Based on Robust Iterative Weighted Similarity Transformation (IWST) Method

The models of geodetic deformation analysis are divided into descriptive and cause-response models [21,22]. Congruence and kinematic models may be classified as descriptive ones, whereas static and dynamic models belong to the cause-response models. The most important task in deformation analysis is to correctly identify unstable points and to isolate them from the set of stable ones. In the literature, various approaches can be found based on congruence models [23], on common adjustment of two measuring epochs based on assumed stable points determined by geoengineering research [24], robust estimates [25,26,27], finite element strain analysis method [22], and others. Most conventional models are based on the method of least squares at all measuring epochs (congruence model, common-adjustment model, finite element strain analysis method). However, a model based on the least squares method is not always realistic. If the data follow a normal distribution, the least squares will yield the most probable values for the estimated parameters. If the assumptions concerning the model are incorrect, due to non-modelled systematic influences (even of a small magnitude), or to correlated observations, the chosen distribution must be modified. Thus, robust variants of standard estimates have been created through which an estimation of parameters is attempted without the influence of the deviation model. This is namely the reason why in the present paper the method of robust deformation estimating is chosen for the needs of analyzing the displacements detected by geodetic measurements.

After the publication of Huber’s paper [28], robust methods have been applied more frequently in deformation analysis. Their basic characteristic is that the parameter estimate is done without a priori assumption about a normal distribution, i.e. the nature of the deviations, which is the main characteristic of the congruence model. The estimates should be close to the true values, even when the data contain gross errors, so that bearing in mind the correct model and the data free of errors they yield almost optimal results [29].

Frequently used robust methods are Iterative Weighted Similarity Transformation (IWST), which was developed at the University of New Brunswick in Canada [26], and Least Absolute Sum [30]. Both methods are based on the application of the S-transformation for detecting the trend of points displacement.

The deformation analysis procedure based on the performed geodetic measurements using the robust estimates is a classic approach based on the IWST method. This method is used for the purpose of estimating the trend of the point displacement and it finds a significant application in numerous studies, among which the best known are Tevatron atomic particle accelerator complex at the Fermilab Laboratory in the USA and automated ALERT monitoring system developed by the Canadian Centre for Geodetic Engineering [31].

The IWST method satisfies the condition of minimum sum of the component moduli of the displacement vector [25,26,27,31,32]. The method is based on the S transformation (Helmert’s similarity transformation):

(1) $${\begin{array}{c} {\hat{d}}^{\left(k\right)}=S^{\left(k\right)}d\\ Q_{\hat{d}}^{\left(k\right)}=S^{\left(k\right)}Q_{d}{\left(S^{\left(k\right)}\right)}^{T}\\ W^{\left(k+1\right)}=diag\left(\begin{array}{cc} \cdots ,w_{S_{i}}^{\left(k+1\right)},\;\cdots & \cdots ,\;0,\;\cdots \end{array}\right) \end{array}\}}_{k=1,\ldots }$$

where $d={\hat{x}}_{2}-{\hat{x}}_{1}$ is the displacement vector, $Q_{d}=\left(Q_{{\hat{x}}_{1}}+Q_{{\hat{x}}_{2}}\right)$ cofactor displacement matrix, $S^{\left(k\right)}=I-H{\left(H^{T}W^{\left(k\right)}H\right)}^{-1}H^{T}W^{\left(k\right)}$ S transformation matrix, I unit matrix, H matrix of datum conditions and W weight matrix.

In the case of two-dimensional geodetic networks, the matrix of datum conditions H has the following form:

$$H={\left[\begin{array}{cc} \begin{array}{cc} \vdots & \vdots \\ 1 & 0 \end{array} & \begin{array}{cc} \vdots & \vdots \\ -{\bar{y}}_{i} & {\bar{x}}_{i} \end{array}\\ \begin{array}{cc} 0 & 1\\ \vdots & \vdots \end{array} & \begin{array}{cc} {\bar{x}}_{i} & {\bar{y}}_{i}\\ \vdots & \vdots \end{array} \end{array}\right]}_{2m\times h}$$

where m is the number of points in the network, h the number of all datum network parameters, and ${\bar{y}}_{i}$ and ${\bar{x}}_{i}$ are adjusted coordinates of the point from the zero-epoch reduced to the network centroid. The first two columns of the matrix represent the translation along the coordinate axes y and x, the third column represents the rotation about the z axis, whereas the fourth one defines the scale [25,26,27,31,33].

Only the points of the basic network can participate in the optimization process (1). Thus, the weights of the points on the object must be zero, because then the points will not be corrected and in this way, they will not participate in the optimization process. In the first iteration of the transformation (k=1) the weight matrix is the unit matrix (W=I). In the subsequent iterations the weights of the basic network points are determined in the following way:

(2) $$w_{S_{i}}^{\left(k+1\right)}=1/\left|{\hat{d}}_{i}^{\left(k\right)}\right|$$

where ${\hat{d}}_{i}$ is the corresponding component of the displacement vector for a point (${\hat{d}}_{y_{i}}$ or ${\hat{d}}_{x_{i}}$). During the iterative optimization process (1), some values of ${\hat{d}}_{i}$ can be very close to zero, causing numerical instability when forming a weight matrix W. Because of this (2) is modified in the following way:

$$w_{S_{i}}^{\left(k+1\right)}=1/(\left|{\hat{d}}_{i}^{\left(k\right)}\right|\;+\;c)$$

where c is the assumed value of the tolerance (for instance, c=0.1 mm). Iterative process (1) is performed until the differences between successively transformed displacement vectors $\left|{\hat{d}}^{\left(k+1\right)}-{\hat{d}}^{\left(k\right)}\right|$ are less than the assumed tolerance c.

For the purposes of testing the stability of the network points, the displacement vector and the corresponding cofactor matrix from the last iteration are used. The stability examination of the network points is performed by applying the single-point test. For this purpose, hypotheses are set:

$$H_{0}:E\left({\hat{d}}_{i}\right)=0\;\mathrm{against}\;H_{a}:E\left({\hat{d}}_{i}\right)\neq 0$$

where ${\hat{d}}_{i}$ is the displacement vector of the i-th point. The test statistics is formed according to the following equation:

(3) $$T_{i}=\frac{{\hat{d}}_{i}{}^{T}Q_{{\hat{d}}_{i}}^{-1}{\hat{d}}_{i}}{h_{i}{\hat{\sigma }}_{0}^{2}}\sim F_{1-\alpha ,\;h_{i},\;f}$$

where ${\hat{d}}_{i}$ is the displacement vector, $Q_{{\hat{d}}_{i}}$ cofactor matrix of the displacement vector, $h_{i}=rank\left(Q_{{\hat{d}}_{i}}\right)$, $f=f_{1}+f_{2}$ total number of degrees of freedom from two measuring epochs and ${\hat{\sigma }}_{0}^{2}=\left(f_{1}{\hat{\sigma }}_{0_{1}}^{2}+f_{2}{\hat{\sigma }}_{0_{2}}^{2}\right)/f$ total a posteriori dispersion coefficient from two measuring epochs.

If $T_{i}\leq F_{1-\alpha ,\;h_{i},\;f}$, the null hypothesis is not rejected, and the point can be regarded as stable. When $T_{i}>F_{1-\alpha ,\;h_{i},\;f}$, the null hypothesis is rejected, and it can be concluded that the point is significantly displaced. Detailed explanations concerning the IWST method can be found in the aforementioned publications [25,26,27,31].

## 3. 2D Deflection Sensor Design

**Figure 1:**
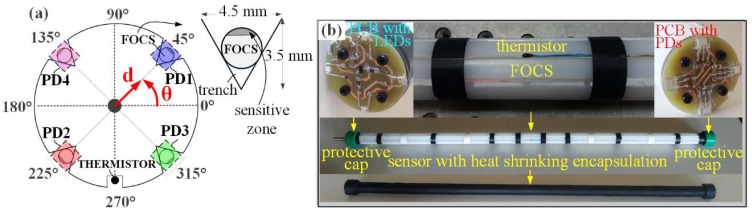
(**a**) Arrangement of the FOCSs and PDs at beam cross section in respect to the reference coordinate system; (**b**) Photo of the fabricated sensor.

The basis of the 2D deflection sensor is a polyamide beam (1020 mm long with 30 mm diameter). On the polyamide beam surface, by using precise tools, five trenches are engraved where four of them have triangular cross sections and are positioned with 90° spacing between them (Figure 1). The fifth trench having a rectangular cross section is positioned in the middle, between two neighboring, arbitrarily selected trenches, and within it the thermistor for temperature monitoring and compensation is placed.

**Figure 2:**
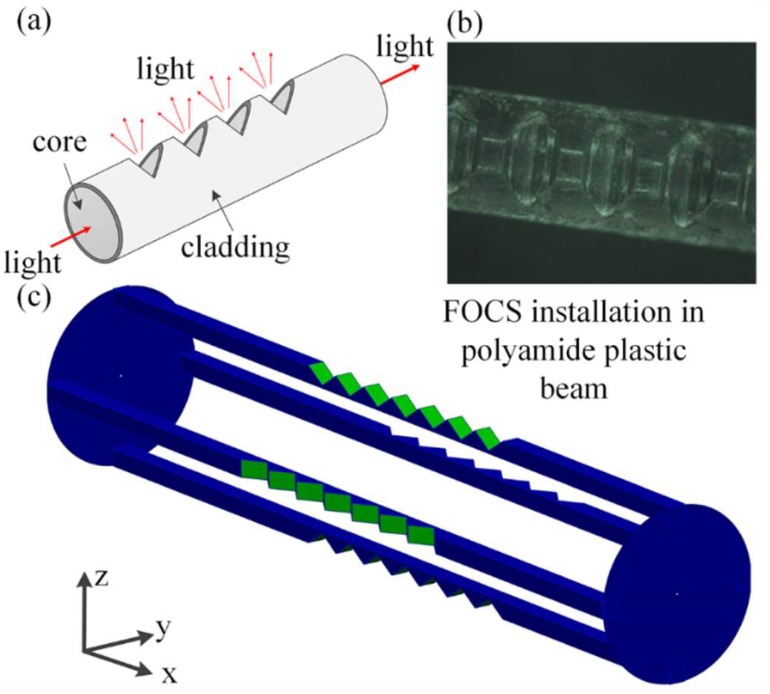
2D deflection sensor based on FOCS, (**а**) Light propagation; (**b**) Photo of teeth; **(c)** FOCSs installation within a polyamide beam.

The four trenches contain four plastic optical fibers, each of 1.5 mm diameter, which constitute 2D deflection sensor (Figure 2). On each optical fiber structural imperfections (teeth) are applied to increase its sensitivity to bending (Figure 2).

**Figure 3:**
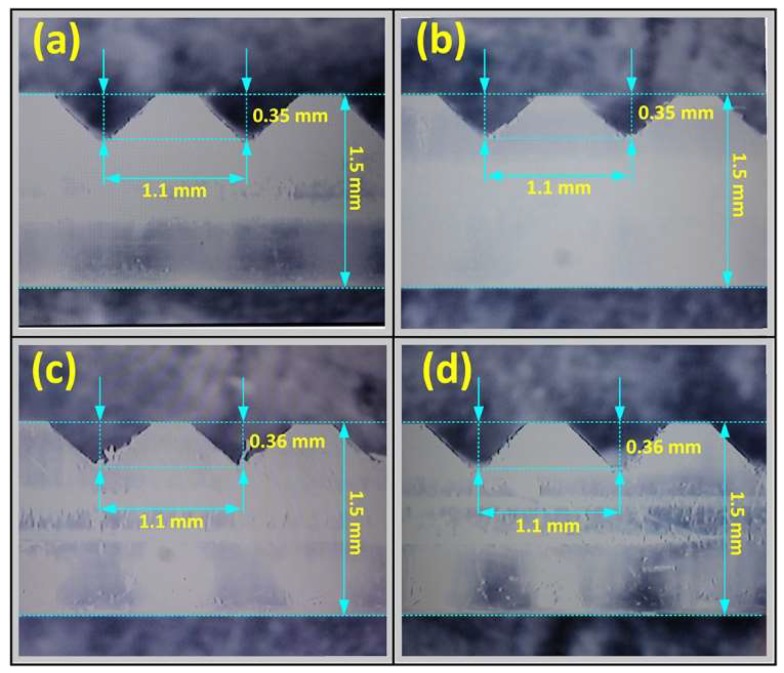
Teeth of the FOCS.

Plastic optical fibers are chosen because of their robustness and low price, as well as simplicity of applying teeth on their surface. The cutting is done by using a precise tool, a Protomat S100 device, produced by LPKF Laser & Electronics AG (Grabsen, Germany). The total number of teeth is 50. Spacing between individual teeth is 1.1 mm, and each of them is 0.35 mm deep (Figure 3). Optical fibers 1 and 3, as well as 2 and 4, are installed mutually parallel within the beam. In this way it is possible that during the deformation of a 2D deflection sensor, by observing the parallel, diametrically opposite optical fibers, one of them detects positive and the other one negative bending.

Considering the size of the engraved trenches any uncontrolled displacement of FOCSs is impossible. To prevent any FOCSs moving outside of the trenches (falling out) all optical fibers are additionally fixed by applying silicon. Also, the whole 2D deflection sensor is protected with the heat-shrinkable encapsulation. FOCSs are installed in such a way that in all trenches their teeth are oriented towards the trench top. FOCSs are, on one side, connected to a light source (LED) and, on the other side, to photodetector (PD). LEDs and PDs are mounted on the circular shaped (30 mm in diameter) printed circuits boards (PCBs) that are screwed to the beam ends (Figure 1b). Both, the LEDs and PDs, are connected to PC via NI USB-6351 card within which the electronics provided for operation of the entire system including FOCSs is integrated and programmed. In this way, the readings of the light intensity are provided in real time. FOCSs are connected to independent LEDs and PDs [20]. The described method of 2D deflection sensor installation makes it possible to perform differential measurements, as well as a higher sensitivity in the measurements of deformations.

2D deflection sensor development, design, calibration and characterization were performed by some of the authors of this manuscript. The values obtained for positional and angular accuracies are ±0.15 mm and ±2.5°, respectively, for the resolution (1σ) of 0.01 mm and 0.33°, respectively. The calibration and characterization were done in a laboratory of the Faculty of Technical Sciences in Novi Sad. Results are given in detail in the study by Bajic et al. [20].

## 4. Test description

**Figure 4:**
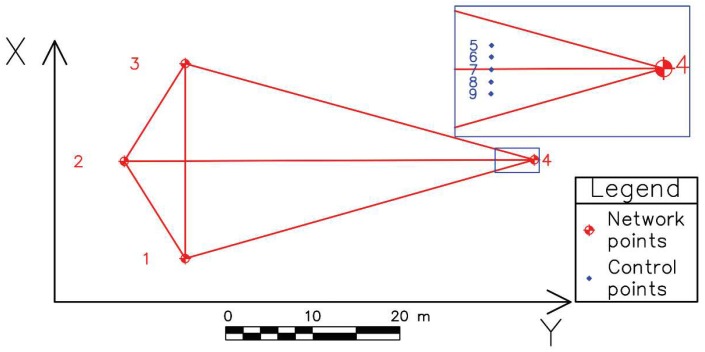
Geodetic micro-network.

**Figure 5:**
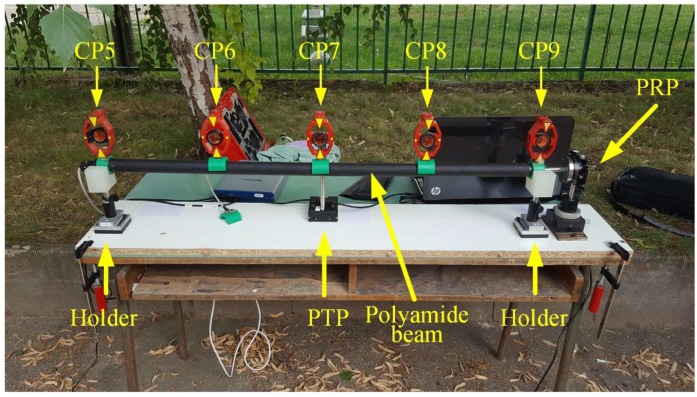
Experimental setup.

The experiment is aimed at comparing two methods of deformation determination, i.e. at comparison of 2D deflection sensor with the geodetic deformation analysis model. To carry out the geodetic measurements a geodetic micro-network consisting of four points is formed (Figure 4). Along a straight line, at distances of about 44 m from point 2 and 3 m from point 4 a setup containing a 2D deflection sensor was placed (Figure 5).

On the 2D deflection sensor there are five mini prisms (control points (CP) 5, 6, 7, 8 and 9) placed at approximately equal mutual distances (Figure 4 and Figure 5). The setup is arranged in the way that the direction of the 2D deflection sensor on which the prisms are is parallel to that defined by points 1 and 3 of the geodetic micro-network. The mini prisms can be observed from points 1, 2 and 3 of the geodetic micro-network, whereas their position is perpendicular to point 2. On a wooden beam, at a 900 mm distance, two holders are tightly fixed. Their role is to prevent any movement of the 2D deflection sensor in the negative direction of the Y axis (Figure 4) and to enable its setting to the optimal height above the wooden beam which is placed on the table. Undesirable 2D deflection sensor movement in the positive direction of the Y axis is not possible because on the other side is placed a precise translation positioner (PTP), PT1/M by Thorlabs (Newton, NJ, USA), that performs the loading directed to the point in the middle of the 2D deflection sensor. The 2D deflection sensor is also at one end tightly fixed to a precise rotation positioner (PRP), PR01/M by Thorlabs, which enables the 2D deflection sensor to rotate and reach a desirable position.

The experiment was performed for comparative determination of the simulated (by PTP) displacement values by applying geodetic measurements and 2D deflection sensor. During the performing geodetic measurements, the following measured quantities were registered: horizontal and vertical angles and oblique lengths. The measurements within the geodetic micro-network are performed in three gyruses with forced centering of the instrument and signal on the tripods, whereas the measurements of the CPs on the sensor are carried out in six independent series, in two gyruses. The CPs are measured simultaneously with three total stations, from points 1, 2 and 3 of the geodetic micro-network. In the zero series the PTP is only leaned on the 2D deflection sensor, without applying additional force, to prevent any movement of the sensor in the positive direction of the Y axis. In every subsequent series the PTP is moved in the negative direction of the Y axis by 1 mm whereby a force action is applied to the middle of the 2D deflection sensor. The 2D deflection sensor approximate position is regulated before the measurements begin by using the PRP so that fibers 3 and 4 are in the horizontal plane, and fibers 1 and 2 are in the vertical plane (Figure 1a). The 2D deflection sensor readings are registered twice, at the beginning and at the end of the geodetic measurements, i.e. after and before the PTP action. Considering the coordinate system of the geodetic micro network, the 2D deflection sensor provides the registration of displacements along the *Y* and *Z* axes. The equipment used in the geodetic measurements is a Leica Geosystems set (Heerbrugg, Switzerland). From points 1 and 3 the measurements are performed with a TCRP1201+ total station with declared accuracies of 1” for direction measurement and 1 mm + 1.5 ppm for length measurement. From point 2 a TS06 total station is used with declared accuracies of 2” for direction measurement and 1.5 mm + 2 ppm for length measurement.

## 5. Measurements results and discussion

The geodetic deformation analysis is performed by applying the IWST method. As already said, iteration process (1) is continued until the differences of the successively transformed displacement vectors are under a value specified for the tolerance c. The value assumed for the tolerance is equal to 0.001 mm.

The results of simulated (by PTP) displacement measurements detected by 2D deflection sensor are presented in Table 1, whereas in Table 2 the measurement results of rotation angle values in the *Y-Z* plane for each individual series are given. The results of the geodetic measurements are presented in Table 3. It should be noted that the applied geodetic measurements measure both, deformed shape and rigid body movements, while 2D deflection sensor measures only deformed shape. As explained earlier, the experiment is performed in five variants with simulated displacements from 1 to 5 mm in steps of 1 mm. Accordingly, in Table 3 the results of the estimated components of the displacement vector in the Y and X axes are presented in five columns, as well as the values of the test statistics (3). In Table 3 in each column the points identified as unstable are indicated (test statistics for (3) exceeds the tabular value of the Fisher distribution depending on the chosen probability and number of degrees of freedom). In all calculations the standard value for the probability is used (1−α=0.95). The measurements in the Z axis are not analyzed, both for 2D deflection sensor and for the geodetic measurements, because the deviations in the Z axis are negligible compared to the registered displacements. Standard deviations of displacement vector components are presented in Table 4. In the first measurement series, using IWST method, significant displacements were not identified because simulated displacements reach the limit of measurement accuracy.

**Figure 6:**
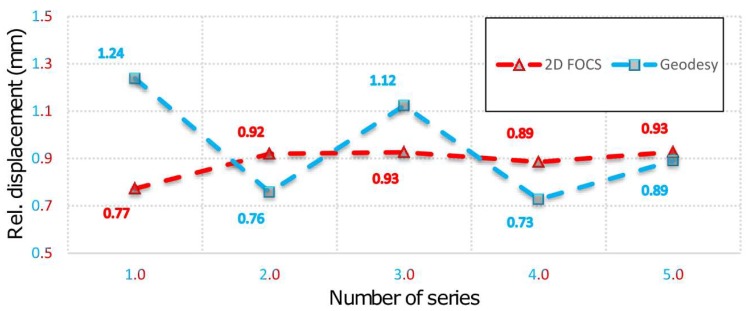
Values of relative displacements registered by 2D deflection sensor and geodetic measurements.

Figure 6 presents the values of the relative displacements expressed in mm registered by 2D deflection sensor and geodetic measurements. It can be noticed that for the simulated displacement of 1 mm (by PTP), the value determined by applying 2D deflection sensor in the first series is equal to 0.77 mm. The mean displacement value in all other series is 0.915 mm with a standard deviation of 0.017 mm which is in accordance with the positional accuracy of 2D deflection sensor equal to ±0.15 mm obtained in the sensor calibration. The average values of the standard deviations of the direction and length measurements with total stations to the CPs are 1.7″ and 0.1 mm, respectively. The average values of the standard deviations of the direction and length measurements with total stations in geodetic network are 1.5″ and 0.05 mm, respectively. These values are consistent with the declared accuracies of used total stations. Maximum values of displacement vector standard deviations for CPs 5 to 9 are 0.69 mm and 0.42 mm, for *Y* and *X* axes respectively. Therefore, measurements using 2D deflection sensor yield more precise results, but it can also be concluded that both technologies have a sub-millimeter accuracy. It can be also noticed, by inspecting Table 2, that only during the first measurement series the value of the measured angle by applying 2D deflection sensor was decreased compared to the zero series. In all other series the value of the measured angle gradually increased to reach a value of 311.9849° in the fifth series.

**Figure 7:**
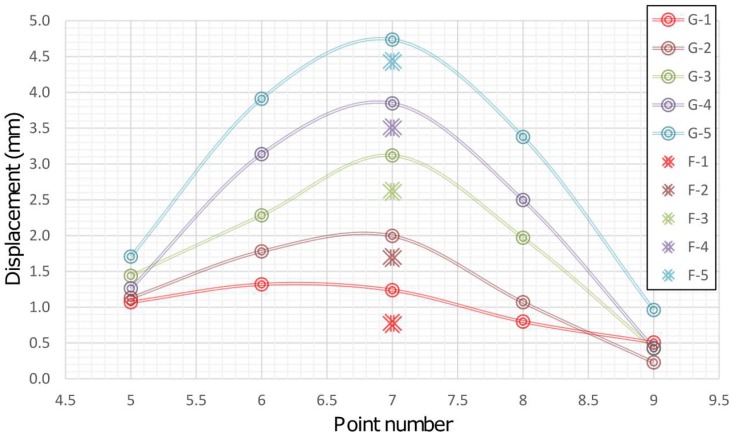
Results of all geodetic and 2D deflection sensor measurements.

**Figure 8:**
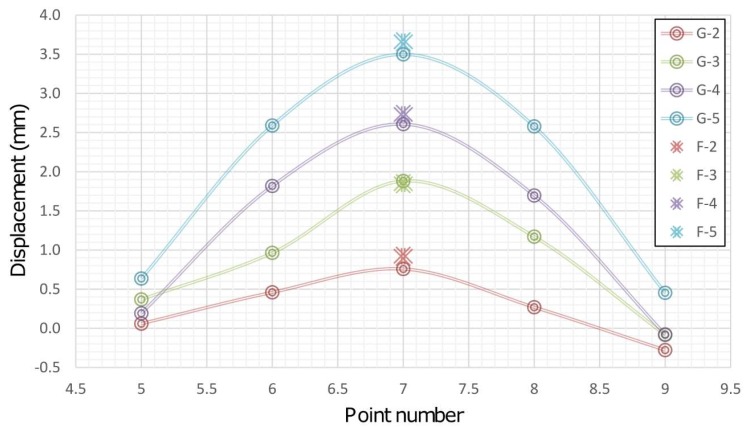
Results of geodetic and 2D deflection sensor measurements without zero series.

**Figure 9:**
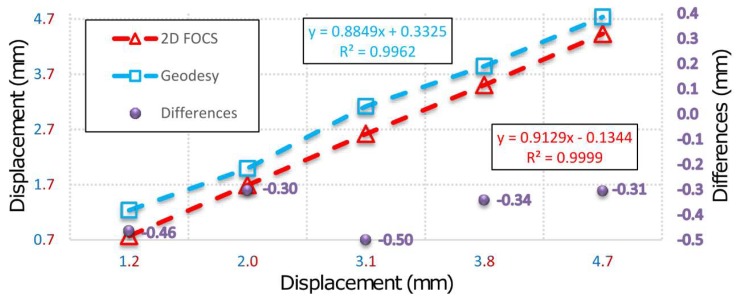
Results and differences for all series of geodetic and 2D deflection sensor measurements for point 7.

**Figure 10:**
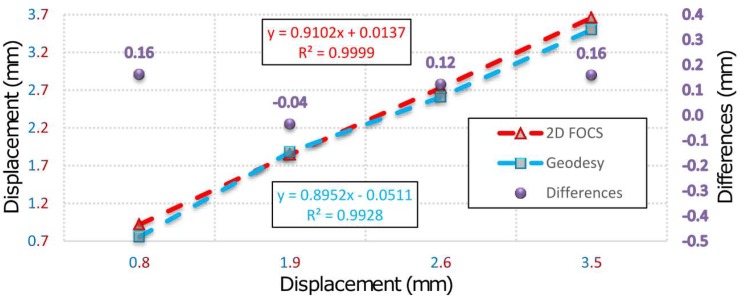
Results and differences of geodetic and 2D deflection sensor measurements for point 7 without zero series.

Based on the above facts, it can be concluded that during the simulation of displacement (by PTP) in the first measurement series there was movement of the beam, which was not registered by 2D deflection sensor. This event is result of the fact that during the action of the PTP the beam was not leaned on the holder which resulted in an "idle motion" of about 0.15 mm in the *X-Y* plane and in a negative rotation of about 3° around the beam rotation axis. In addition, the insight into the movements of the CPs 5 and 9 determined by applying geodetic measurements indicates that the beam also rotated in the *X-Y* plane. This happened because the beam was rigidly bound to the PRP in the immediate neighborhood of CP 9 so that the base rotation point was also a contact between the PTP and the beam. In the first measurement series an error in the experimental setup was evidently manifested. The error occurred first due to the beam rotation, but there is a possibility that the beam was also influenced to a small extent by the stability of the holders, PRP and PTP, basis of the mini prisms on which the PTP was leaned and by the thickness and irregular shape of the heat-shrinkable encapsulation. Since the stability of the entire measurement system was achieved after the first measurement series, the analysis of the collected data is done with and without the zero series. Figure 7 concerns all measurement series, whereas in Figure 8 the zero series is excluded. G (1-5) represent geodetic measurements and F (1-5) represent 2D deflection sensor measurements. An inspection of Figure 7 and Figure 8 shows that by eliminating the zero series the error which appeared during the measurements in the first series is also eliminated to a large extent. Measured results and differences for point 7 by applying 2D deflection sensor and geodetic measuring are presented in Figure 9 (all series included) and Figure 10 (zero series excluded).

## 6. Conclusions

In the present paper a direct comparison of a simple, low-cost 2D deflection sensor for deformation determination and geodetic measurements is done. If only the measurements performed with 2D deflection sensor were considered, it would not be possible to establish that during the measurement process the beam rotation occurred which is confirmed by the geodetic measurements. However, an inspection of the 2D deflection sensor measurements clearly indicates a displacement of the sensor by about 0.15 mm during the motion in the first measurement series, which was not registered by 2D deflection sensor. This is an additional information whereby the effect of the 2D deflection sensor rotation, detected by the geodetic measurements, is also confirmed. The method of measuring deformations by applying an integrated system, consisting of 2D deflection sensor and total stations, would be applicable in the continuous monitoring of arch dams or bridges. It would be necessary to install an optimal number of 2D deflection sensors evenly distributed over the longitudinal section of the object and based on their measurements it would be possible to approximate the geometry of the entire span of the bridge or arch dam. Rigidly stabilized the geodetic reflectors (prisms) would be necessary to place at the support points of the bridge span (pillars, dilatations, etc.), or in the case of an arch dam, on the dam, nearby the touch point of the dam with the surrounding terrain, to monitor absolute deformations of the mentioned building structures. Based on the study described in the present paper it can be concluded that the proposed system with a 2D deflection sensor can be successfully used in the monitoring of deformations. In addition, based on all facts presented in the paper it is possible to reach the conclusion that by integrating several distinct sensor types more complete and more reliable results of deformation measurements can be obtained and, consequently, make the correct decisions in the potential SHM process.

## Figures and Tables

**Table 1:** Results of measuring displacements by applying 2D deflection sensor.

Point Number	0-1	0-2	0-3	0-4	0-5
	$\hat{d}\left(mm\right)$	$\hat{d}\left(mm\right)$	$\hat{d}\left(mm\right)$	$\hat{d}\left(mm\right)$	$\hat{d}\left(mm\right)$
7	0.77	1.69	2.62	3.50	4.43

**Table 2:** Results of reading the values of angles by applying 2D deflection sensor.

Point Number	0	1	2	3	4	5
	Angle (°)	Angle (°)	Angle (°)	Angle (°)	Angle (°)	Angle (°)
7	309.2964	306.2564	309.5777	310.8704	311.2057	311.9849

**Table 3:** Geodetic measurement results.

Point Number	0-1		0-2		0-3		0-4		0-5	
	${\hat{d}}_{y}$	$T_{i}$	${\hat{d}}_{y}$	$T_{i}$	${\hat{d}}_{y}$	$T_{i}$	${\hat{d}}_{y}$	$T_{i}$	${\hat{d}}_{y}$	$T_{i}$
	${\hat{d}}_{x}$		${\hat{d}}_{x}$		${\hat{d}}_{x}$		${\hat{d}}_{x}$		${\hat{d}}_{x}$	
	(mm)		(mm)		(mm)		(mm)		(mm)	
1	0.00	0.14	0.00	0.00	0.01	0.03	0.00	0.02	0.00	0.00
	−0.12		0.00		−0.07		0.05		0.00	
2	−0.05	0.05	0.00	0.00	−0.02	0.00	0.03	0.02	0.01	0.00
	0.00		0.01		0.00		0.00		0.00	
3	0.00	0.12	0.00	0.00	0.02	0.03	0.02	0.02	0.00	0.00
	0.12		−0.01		0.07		−0.04		−0.01	
4	0.07	0.01	−0.03	0.00	−0.01	0.00	−0.12	0.04	−0.04	0.00
	0.00		0.00		0.00		0.00		0.00	
5	−1.04	1.60	−1.13	1.43	−1.33	2.80	−1.28	1.71	−1.70	3.23
	0.29		−0.09		0.55		0.10		0.13	
6	−1.25	2.43	−1.78	3.50^a^	−2.22	5.96^a^	−3.12	10.15^a^	−3.90	16.89^a^
	0.39		−0.04		0.49		0.19		0.14	
7	−1.23	1.90	−1.95	4.83^a^	−3.09	10.71^a^	−3.85	15.48^a^	−4.75	24.90^a^
	0.13		−0.45		0.40		0.13		−0.05	
8	−0.74	0.87	−1.03	1.42	−1.95	4.49^a^	−2.50	6.58^a^	−3.35	12.52^a^
	0.25		−0.29		0.38		−0.08		0.20	
9	−0.28	0.78	−0.32	0.11	−0.30	0.47	−0.46	0.31	−0.95	1.31
	0.45		−0.02		0.36		0.20		0.32	

^a^ The test statistics exceeds the critical value $F_{0.95,\;2,70}=3.13$.

**Table 4:** Standard deviations of displacement vector components.

Point Number	0-1	0-2	0-3	0-4	0-5
	$\sigma_{{\hat{d}}_{y}}$	$\sigma_{{\hat{d}}_{y}}$	$\sigma_{{\hat{d}}_{y}}$	$\sigma_{{\hat{d}}_{y}}$	$\sigma_{{\hat{d}}_{y}}$
	$\sigma_{{\hat{d}}_{x}}$	$\sigma_{{\hat{d}}_{x}}$	$\sigma_{{\hat{d}}_{x}}$	$\sigma_{{\hat{d}}_{x}}$	$\sigma_{{\hat{d}}_{x}}$
	(mm)	(mm)	(mm)	(mm)	(mm)
1	0.09	0.12	0.20	0.03	0.09
	0.25	0.11	0.27	0.27	0.17
2	0.16	0.11	0.22	0.20	0.16
	0.05	0.23	0.01	0.02	0.14
3	0.14	0.14	0.23	0.26	0.14
	0.25	0.28	0.27	0.27	0.26
4	0.46	0.49	0.33	0.50	0.48
	0.04	0.04	0.01	0.01	0.05
5	0.64	0.67	0.68	0.69	0.67
	0.38	0.40	0.41	0.42	0.40
6	0.64	0.67	0.68	0.69	0.67
	0.38	0.40	0.41	0.42	0.40
7	0.64	0.67	0.68	0.69	0.67
	0.38	0.40	0.41	0.42	0.40
8	0.64	0.67	0.68	0.69	0.67
	0.38	0.40	0.41	0.42	0.40
9	0.64	0.67	0.68	0.69	0.67
	0.38	0.40	0.41	0.42	0.40
